# Case Report: Minimally invasive management of suspected active bleeding from intercostal vessel after axillary thoracotomy ventricular septal defect repair: an application of Foley catheter

**DOI:** 10.3389/fcvm.2025.1511221

**Published:** 2025-05-27

**Authors:** Yunfei Tian, Erjia Huang, Mengdi Zhang, Jinzhe Fan, Wei Li, XiaoHui Yang, Wei Su, Xicheng Deng

**Affiliations:** ^1^ Heart Center, The Affiliated Children’s Hospital of Xiangya School of Medicine, Central South University, Changsha, China; ^2^ Department of Cardiovascular Surgery, The Second Xiangya Hospital, Central South University, Changsha, China; ^3^ Clinical College, Xiangnan University, Chenzhou, China; ^4^ Department of Pediatrics, Affiliated Hospital of Xiangnan University, Chenzhou, China

**Keywords:** Foley catheter, chest drain, postoperative bleeding, ventricular septal defect, minimally invasive, axillary thoracotomy

## Abstract

The right axillary thoracotomy, an alternative approach for selected open-heart procedures, offers aesthetic advantages. However, intercostal vessel injury is a potential postoperative complication that can lead to major bleeding. Herein, we report a case of postoperative active bleeding from intercostal vessel injury after right axillary thoracotomy for ventricular septal defect repair. Hemorrhage was successfully halted by compressing the suspected bleeding site with a Foley catheter inserted through the chest wall. This case demonstrates a simple management method of active intercostal vascular bleeding after cardiac operation. It may be an appropriate option in selected postoperative patients with a high index of suspicion of intercostal vascular bleeding after operation via a thoracotomy approach.

## Introduction

Recent years have witnessed the advent of minimally invasive surgical techniques, including interventional procedures, robotics, and axillary approaches. The right axillary thoracotomy is an alternative approach for selected cardiac procedures requiring cardiopulmonary bypass. This technique offers an aesthetic advantage since the incision is hidden beneath the arm (1, 2). However, intercostal vessel injury is a potential postoperative complication that can lead to major bleeding. The conventional management of active hemorrhage involves open exploration to locate and repair the bleeding site. This approach, while effective, is invasive and can be associated with higher morbidity and longer recovery times.

Achieving hemostasis through balloon compression has rarely been documented. We present a case of active intercostal bleeding following a right axillary thoracotomy, which was controlled by inserting a Foley catheter to compress the presumed bleeding site. This minimally invasive technique offers a less invasive alternative to conventional re-exploration, potentially reducing morbidity and recovery time. We have reported this case in line with the SCARE criteria (3–5).

## Presentation of case

A 5-year-old child was admitted to our institution for repair of a ventricular septal defect (VSD). The operation was performed under general anesthesia and extracorporeal circulation via a right axillary thoracotomy at the fourth intercostal space. Following closure of the VSD, intermittent 2:1 conduction was observed upon removing the cross clamp. A temporary pacing wire was inserted into the right ventricle and passed through the sixth intercostal space. During chest closure, active bleeding was noted at the site where the pacing wire penetrated the chest wall. Prompt action was taken, including the placement of a purse-string suture to achieve hemostasis. Subsequent verification of the surgical and drain incision sites confirmed no further active bleeding.

Upon returning to the ICU following surgery, the patient exhibited a pale complexion and lips. The heart rate was alarmingly high, ranging from 140 to 160 beats per minute, while blood pressure showed significant instability, fluctuating between 53 and 60 mmHg systolic and 32–36 mmHg diastolic. Notably, there was substantial drainage from the right pleural drainage tube, with approximately 260 ml collected within the first three hours post-operation. Concurrently, the central venous pressure (CVP) measured 4 cmH2O, and the urine output was approximately 35 ml per hour.

**Figure 1:**
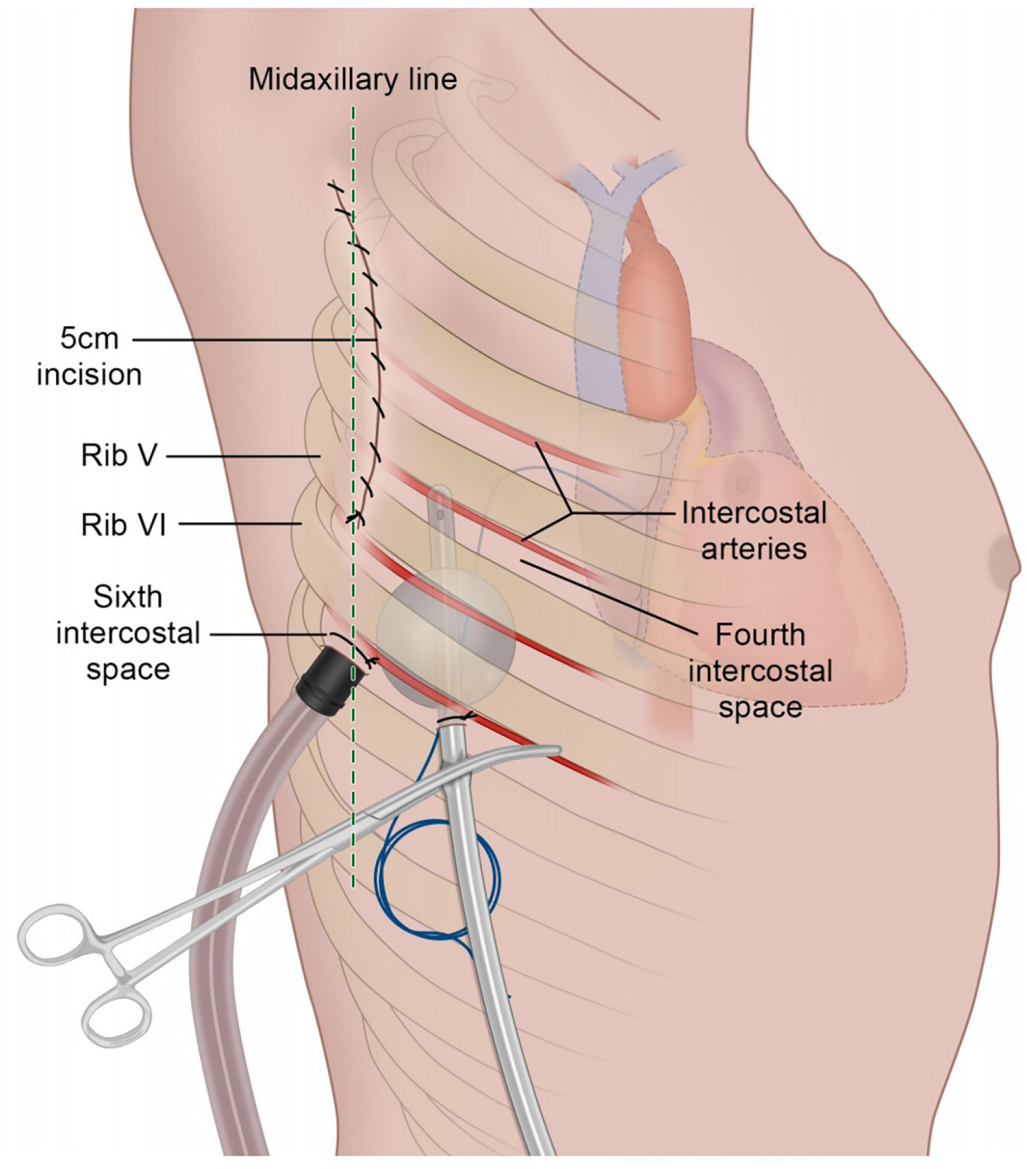
Foley catheter compresses the bleeding site.

Based on the clinical manifestations of low blood pressure, elevated heart rate, and substantial thoracic drainage volume, along with the observation that arterial blood typically exhibits a jet-like spurt pattern while the drainage fluid appears bright red with relatively slow bleeding velocity and low pressure, we hypothesize that the persistent hemorrhage may result from intercostal venous vascular injury caused by pacemaker lead penetration, leading to continuous bleeding.This was despite a meticulous hemostatic examination during the closure of the chest cavity. Considering the patient's age and the technical challenges associated with quickly locating and suturing the bleeding site, re-exploring the chest could potentially increase the risk of secondary injury. In light of these considerations, we promptly prepared for bedside exploration and opted for a Foley catheter to achieve compression hemostasis in the meantime. Selecting a 10-French Foley catheter based on the patient's age, we infused 5 ml of normal saline as per the catheter's specifications. The catheter was then gently withdrawn and clamped against the chest wall to effectively compress the bleeding site (see Figure 1). This intervention led to a gradual reduction in the drainage volume. Subsequent echocardiography and bedside chest radiography were conducted to rule out the possibility of cardiac tamponade resulting from obstruction of the drainage tube. The patient's heart rate stabilized between 100 and 140 beats per minute, and blood pressure improved to a stable range of 80–90 mmHg systolic and 50–60 mmHg diastolic. The 24-h drainage volume decreased to approximately 20 ml, with CVP values between 5 and 8 cmH2O, red blood cell count (RBC) at 4.36 × 106/m^3^, hemoglobin (HGB) at 128 g/L, and platelet count (PLT) at 150,000/m^3^. These vital signs indicated that the Foley catheter had successfully compressed the bleeding site.

After confirming the absence of active bleeding and the restoration of sinus rhythm, the drainage tube and pacemaker lead were safely removed on the second postoperative day. At the three-month follow-up, repeat imaging revealed no significant abnormalities, and the child's overall condition was favorable. Informed consent for the publication of this case was obtained from the parents.

## Discussion

Open heart surgery via right axillary thoracotomy is becoming the choice of approach in selected cases (6, 7). The utilization of a right axillary thoracotomy for certain cardiac procedures offers advantages such as reduced trauma and quicker recovery times compared to median sternotomy (8). However, it is also associated with specific risks, including an increased likelihood of intercostal vessel injury and subsequent bleeding due to the proximity of the incision to these structures.

The placement of chest drains tubes following open heart surgery is standard practice. Careful consideration was given to the placement of the drain, with efforts made to puncture along the upper edge of the lower rib, followed by meticulous checks to ensure no active bleeding at the puncture site.

The insertion of temporary epicardial pacing wires can further complicate matters, especially in cases where positioning the pacing lead proves challenging. While pacing leads are not universally utilized in all cardiac procedures, they are employed in scenarios similar to the one presented in this case report. Not like the placement of a chest drain, it is difficult to confirm, in relation to the intercostal vessels, the site where the lead penetrates inside out through the chest wall, hence increasing the risk of injury and bleeding of intercostal vessels, which was the case for the present patient.

Swift control of hemorrhage is critical following vascular injury to restore blood volume and prevent hemodynamic compromise. External compression of the wound represents a rapid means of achieving hemostasis and limiting ongoing blood loss. The insertion of a Foley catheter with subsequent inflation of the balloon can effectively tamponade bleeding from an injured intercostal vessel through direct compression at the hemorrhage site. The selection of the 10-French catheter was based on three main factors: 1. Pediatric intercostal space dimensions, which average 4–6 mm in 5-year-olds. 2. Balloon inflation capacity, with 5 ml providing optimal radial pressure. 3. Minimizing trauma while ensuring effective tamponade. For adult applications, we would recommend upsizing to 12–14-French catheters with proportionally larger balloon volumes (8–10 ml).

In this case, while re-exploration was contemplated, it was not immediately pursued to minimize trauma to the patient. Instead, a presumption of the hemorrhage site was made based on clinical evidence. The surgical incision and drain port had been confirmed with no bleeding, yet the pacing lead site was found with bleeding during chest closure even though hemostasis was achieved at once. Therefore, the postoperative bleeding was highly suspected to be from the pacing lead site. Balloon tamponade was successfully employed to achieve hemostasis without the need for further surgical intervention. This minimally invasive approach not only effectively controlled bleeding but also minimized additional trauma and expedited the patient's recovery. We have compiled a comparative table summarizing this minimally invasive approach, which is presented as Table 1 at the end of the article.

**Table 1:** Comparison of different methods for intercostal vessel injury management.

Management option	Indication	Procedure description	Advantages	Risks/complications
Foley Catheter (intercostal compression)	Small to moderate bleeding in stable patients	Insert Foley catheter into pleural space to provide localized compression.	Non-invasive, bedside, minimal anesthesia.	Displacement, infection, incomplete hemostasis.
Surgical Re-exploration	Severe or uncontrolled bleeding	Thoracotomy for direct visualization and vessel ligation/cauterization.	Definitive control; manages complex injuries.	Infection, bleeding, lung damage, prolonged recovery.
Endovascular techniques	Moderate to severe bleeding, high-risk surgical patients	Embolization or stenting to occlude bleeding vessels.	Minimally invasive, effective for unstable patients.	Embolization failure, re-bleeding, catheter-related issues.
Chest tube with hemostasis	Moderate bleeding with hemothorax	Chest tube inserted for blood evacuation and clot formation.	Simple, helps control bleeding while monitoring.	Recurrent bleeding, infection, tube dislodgement.

Similar applications of balloon compression for damage control have been documented across various medical specialties, including in cases of hepatic, abdominal, orthopedic, and previous cardiac surgical trauma, and multiple heel and rib fractures and multiple rib fractures, and similar to the present case for bleeding from intercostal vascular injuries after cardiac surgery (9–14).

**Figure 2:**
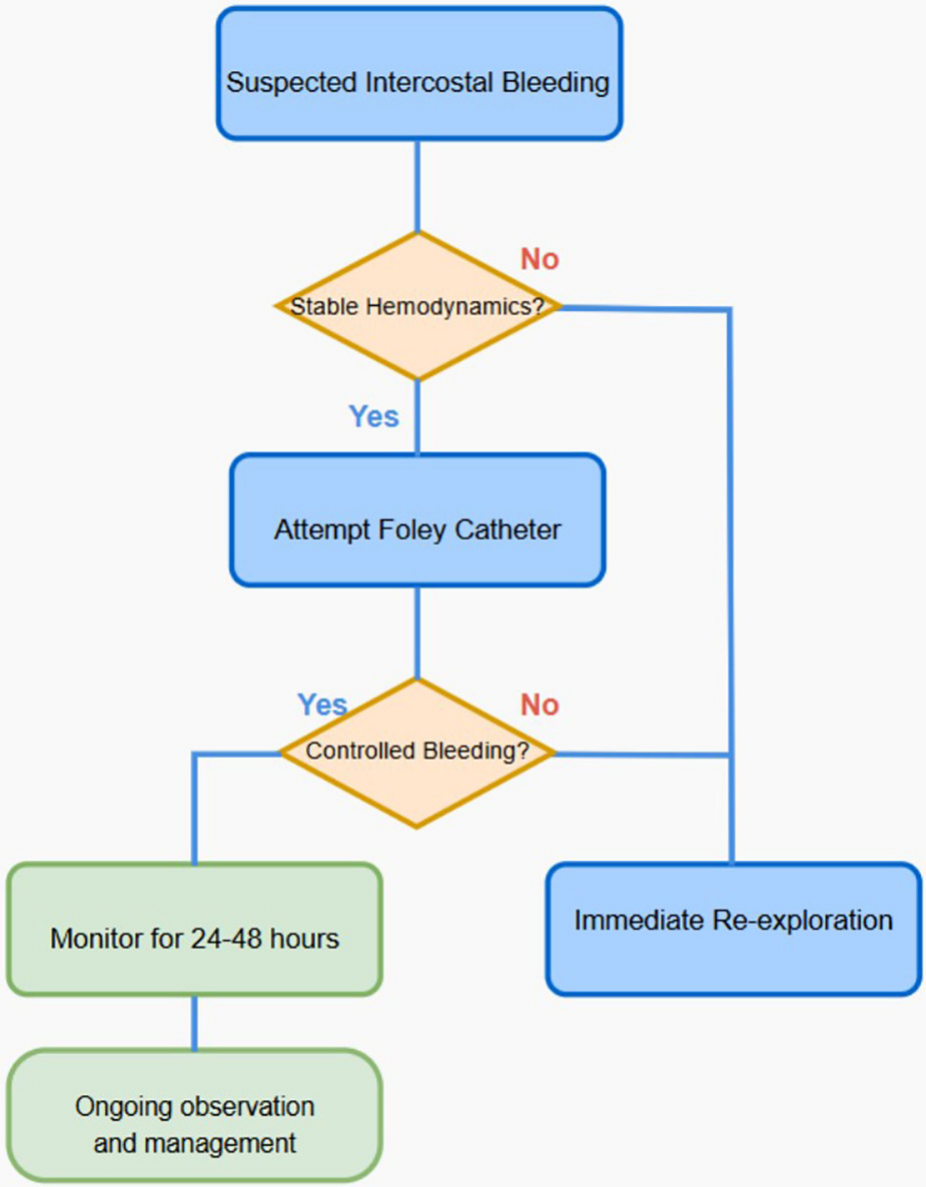
Evaluating the procedure of balloon compression using a Foley catheter.

This case highlights the critical role of vigilant postoperative monitoring and the strategic application of innovative techniques, such as Foley catheter compression, in effectively managing complications like active intercostal bleeding following cardiac surgery via a thoracotomy approach. To further enhance the clarity and applicability of our methodology, we have added a new flowchart detailing the patient selection criteria as follows Figure 2. Figure 2 flowchart detailing the patient selection for balloon compression using a Foley's catheter.

## Conclusion

Our report highlights a straightforward management approach for active intercostal vascular bleeding following cardiac surgery. This method may represent a suitable option in carefully selected postoperative patients where there is a high suspicion of intercostal vascular bleeding via a thoracotomy approach. By employing balloon tamponade, clinicians can effectively address hemorrhage while minimizing the need for reoperation, thereby promoting patient recovery and reducing the risk of further complications.

## Data Availability

The original contributions presented in the study are included in the article/Supplementary Material, further inquiries can be directed to the corresponding authors.
